# Consistent long-term practice leads to consistent improvement: Benefits of self-managed therapy for language and cognitive deficits using a digital therapeutic

**DOI:** 10.3389/fdgth.2023.1095110

**Published:** 2023-04-11

**Authors:** Hantian Liu, Claire Cordella, Prakash Ishwar, Margrit Betke, Swathi Kiran

**Affiliations:** ^1^ Department of Computer Science, College of Arts and Sciences, Boston University, Boston, MA, United States; ^2^ Center for Brain Recovery, Boston University, Boston, MA, United States; ^3^ Department of Electrical and Computer Engineering, College of Engineering, Boston University, Boston, MA, United States

**Keywords:** aphasia, stroke, technology, rehabilitation, dosage, therapy, data science

## Abstract

**Background:**

Although speech-language therapy (SLT) is proven to be beneficial to recovery of post-stroke aphasia, delivering sufficiently high amounts of dosage remains a problem in real-world clinical practice. Self-managed SLT was introduced to solve the problem. Previous research showed in a 10-week period, increased dosage frequency could lead to better performance, however, it is uncertain if dosage still affects performance over a longer period of practice time and whether gains can be seen following practice over several months.

**Objective:**

This study aims to evaluate data from a health app (Constant Therapy) to investigate the relationship between dosage amount and improvements following a 30-week treatment period. Two cohorts of users were analyzed. One was comprised of patients with a consistent average weekly dosage amount and the other cohort was comprised of users whose practice had higher variability.

**Methods:**

We conducted two analyses with two cohorts of post-stroke patients who used Constant Therapy. The first cohort contains 537 “consistent” users, while the second cohort contains 2,159. The 30-week practice period was split into three consecutive 10-week practice windows to calculate average dosage amount. In each 10-week practice period, patients were grouped by their average dosage into low (0–15 min/week), medium (15–40 min/week) and moderate dosage (greater than 40 min/week) groups. Linear mixed-effects models were employed to evaluate if dosage amount was a significant factor affecting performance. Pairwise comparison was also applied to evaluate the slope difference between groups.

**Results:**

For the consistent cohort, medium (*β *=* *.002, *t*_17,700_ = 7.64, *P* < .001) and moderate (*β *=* *.003, *t*_9,297_ = 7.94, *P* < .001) dosage groups showed significant improvement compared to the low dosage group. The moderate group also showed greater improvement compared to the medium group. For the variable cohort in analysis 2, the same trend was shown in the first two 10-week windows, however, in weeks 21–30, the difference was insignificant between low and medium groups (*β *=* *.001, *t* = 1.76, *P* = .078).

**Conclusions:**

This study showed a higher dosage amount is related to greater therapy outcomes in over 6 months of digital self-managed therapy. It also showed that regardless of the exact pattern of practice, self-managed SLT leads to significant and sustained performance gains.

## 1. Introduction

Stroke is the most common disease that causes serious neurological disorders (1). Every year, over 795,000 people in the United States have a stroke, and aphasia or other communication disorders develop in approximately one-third of cases (2, 3). Compared to other patients, patients with aphasia are facing higher mortality and a higher degree of functional limitation, communication limitation, and social isolation (4, 5), making the need for effective rehabilitative approaches especially acute.

Previous research has shown that speech-language therapy (SLT) benefits functional language, language comprehension (listening and reading), and language production (speaking and writing) (6–14). Results also indicated that therapy at high intensity, high dosage, or over a longer period might be more beneficial compared to lower-intensity therapy (6). Moreover, high-intensity SLT over a short period appeared to help participants' language use in daily life and reduced the severity of their aphasia. However, high-intensity treatments might be less acceptable than less intensive therapy schedules for patients, as indicated by a significantly greater drop-out rate for higher-intensity regimens (6). Besides acceptability, there was also the problem of delivering sufficiently high therapy doses to patients in the real world, where practical realities (e.g., reimbursement caps, difficulties with mobility and travel, geographic isolation) placed severe limits on the amount of therapy actually received. National statistics available from the American Speech-Language-Hearing Association (ASHA) demonstrated a substantial reduction in the frequency and amount of SLT by the time patients had been discharged from acute or inpatient settings to community-based outpatient settings (15–17). A recent study of dosage amounts in a U.S.-based outpatient setting reported a median total therapy dosage of just 7.5 h for individuals with post-stroke aphasia (18). Similarly, another study of access to outpatient post-stroke rehabilitation services found that the average total dosage of outpatient SLT was 8 h total in the year following an individual's stroke (19). These average numbers were far from the number of hours of therapy recommended for high-intensity SLT. In fact, meta-analytic reviews have characterized high-intensity SLT protocols as providing total therapy dosages between 27 and 208 h, with positive effect studies tending to provide at least 50 total hours of therapy (6, 20).

Enabling patients to engage in in-home practice through computerized or app-based therapeutic programs could help patients to get more sufficient amounts of therapy and meet the dosage requirements of high-intensity SLT (13). Digital SLT interventions have been used as part of a treatment protocol in the form of smartphone, tablet, or computer-based programs. Some of these programs are entirely self-managed, meaning that patients can determine their own therapy schedule (14, 21–23). By giving patients the freedom to determine their practice schedule, researchers can access a wide range of practice frequencies, amounts and overall practice patterns from patient to patient. This variability provides a unique opportunity to probe practice-response relationships in SLT *via* dose articulation studies, which are a necessary first step toward the ultimate goal of establishing optimal dosage recommendations for SLT interventions (23, 24).

Recent efforts by Cordella et al*.* analyzed retrospectively collected data to evaluate the optimal dosage of interventions. In this study, the authors directly compared different dosage amounts of the same intervention in the context of self-managed digital therapies (23). This study focused on the relationship between the varied dosage frequency and the performance outcome across 13 different skill domains following a 10-week period of self-managed digital SLT. The results showed that higher dosage frequency groups (e.g., four or five times per week) achieved greater improvement vs. lower ones (e.g., once or twice per week) across all domains and also within a majority of individual subdomains. However, the definition of dosage in the Cordella et al*.* study is primarily the median number of days in a week patients practice, which is only one parameter to evaluate overall dosage (25). Other ways to calculate dosage have included session duration, total intervention duration, and total number of sessions administered (24–32). Moreover, it is not clear that 10 weeks is a sufficient duration of language therapy, especially in chronic survivors. Consequently, it is useful to evaluate improvements over a longer time period than 10 weeks, by which it would be possible to discover potentially more nuanced relationships between dosage and performance.

The goal of this study was to examine real-world therapy data to investigate the relationship between dosage amount, and midpoint and cumulative improvements following a 30-week treatment period using the Constant Therapy app. There were two main objectives of the current study. First, we investigated whether greater average weekly dosage—defined as number of minutes per week—led to greater performance gains over a 30-week period in a cohort of consistent users who practiced approximately the same average amount week to week. Second, in a larger cohort of more variable users we investigated the effect of weekly therapy dosage on performance outcomes across three consecutive 10-week intervals for a total of 30 weeks (i.e., 6 months). The two cohorts were denoted as consistent cohort and variable cohort. We hypothesized that in both analyses, greater practice amount would lead to better performance outcomes. Prior work has shown that during the first 10-week period of therapy, higher dosage frequency groups improved more compared to lower ones across all domains (23). Therefore, we hypothesized that such trend would persist in longer-term therapy that was practiced beyond 10 weeks.

## 2. Methods

### 2.1. Participants

**Figure 1:**
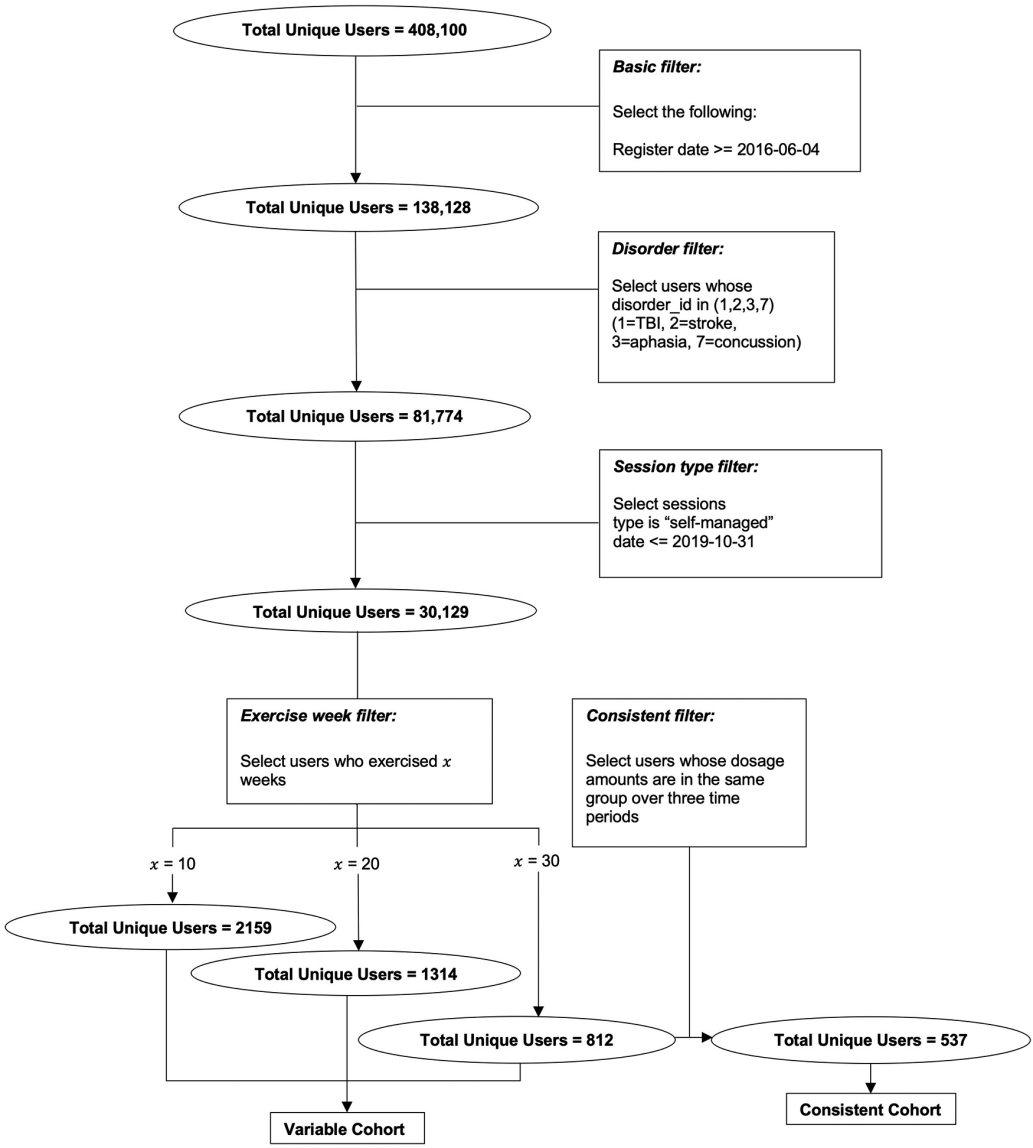
Flow chart of the data filtering procedure that results in the two cohorts for which analysis 1 and 2 are conducted, respectively.

Data used in this study are from patients who used the Constant Therapy app between March 2016 and July 2020. 30,129 unique users who reported having had a stroke with resultant speech, language, and cognitive deficits were included in the analyses with their consent to using exercise and performance outcome data for research purposes. In order to evaluate the performance of longer-term therapy, a smaller number of users were filtered using criteria described in detail below. Overall, all users were engaged in the app for more than 10 weeks in order for their data to be included in the analyses. As described above, in the first cohort, 537 users practiced 30 weeks of consistent therapy (i.e., consistent cohort). In the second, variable cohort, the number of users differed among time periods. 2,159 patients are considered in the first 10 weeks, 1,314 in the second 10 weeks, and 812 in the last 10 weeks. The filtering procedure flowchart is shown in Figure 1 to describe how we select the users from the whole population in the database. Note that all sessions we selected are self-managed sessions, which means no interference is made by any other individual including clinicians or Constant Therapy support team. Demographic details regarding participants are provided in Table 1 after the filtering criteria are described.

**Table 1:** Summary statistics of the consistent cohort (*N =* 1,448).

Characteristics	Overall (*N =* 1,448)	0–15 min/week	15–40 min/week	>40 min/week
Age, mean (SD)	63.13 (13.68)	62.43 (13.98)	63.10 (13.86)	64.74 (12.69)
Baseline domain score, mean (SD)	0.32 (0.20)	0.33 (0.22)	0.30 (0.19)	0.33 (0.18)
**Sex, *n* (%)**				
Male	820 (56.6)	423 (56.0)	193 (55.0)	204 (59.8)
Female	628 (43.4)	333 (44.0)	158 (45.0)	137 (40.2)
Other	0 (0)	0 (0)	0 (0)	0 (0)
**Chronicity, *n* (%)**				
Acute (<6 months)	705 (48.7)	364 (48.1)	169 (48.1)	172 (50.4)
Chronic (>6 months)	743 (51.3)	392 (51.9)	182 (51.9)	169 (49.6)

*N* (patients) = 537.

### 2.2. Constant therapy program

**Figure 2:**
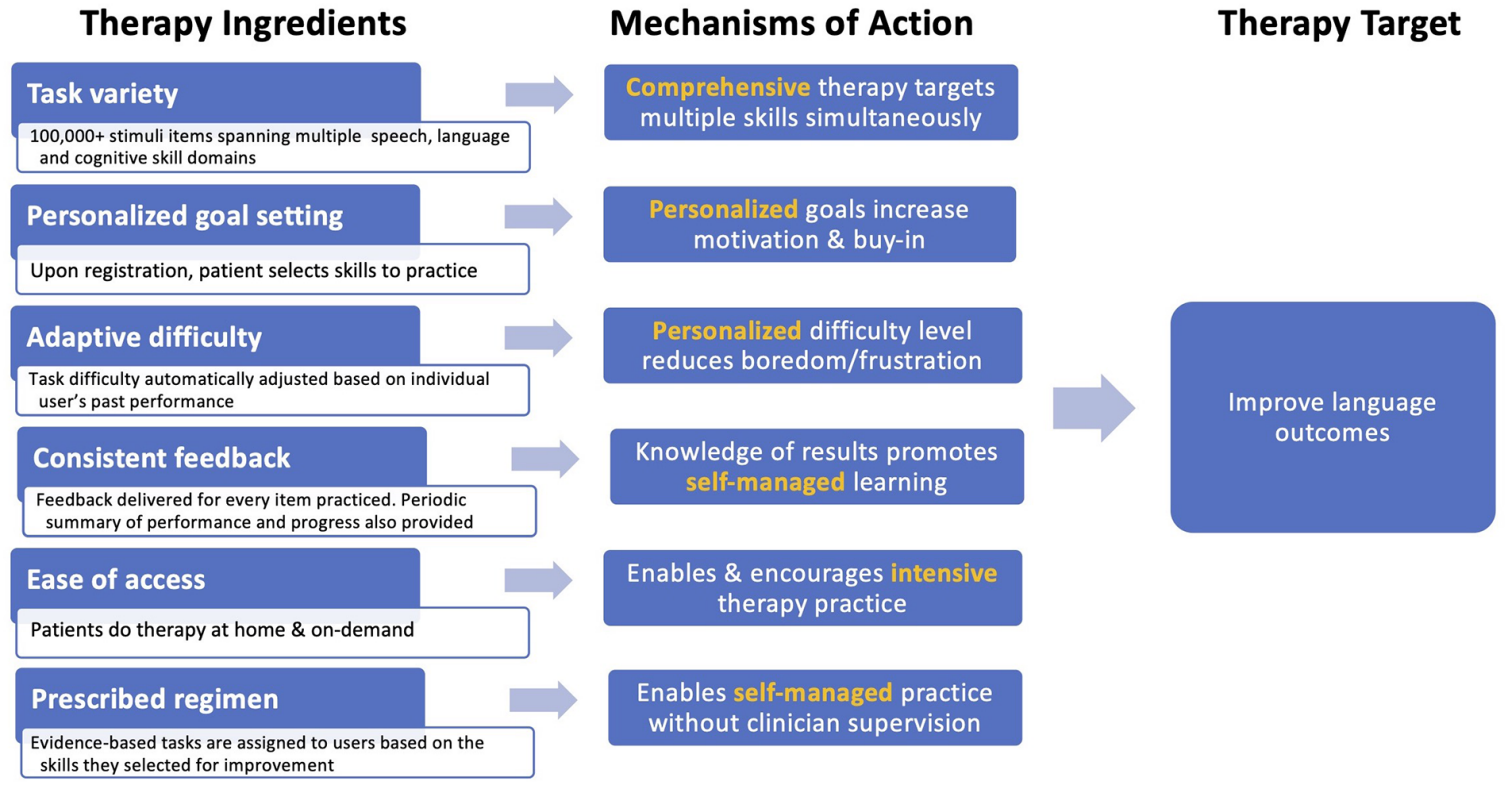
Ingredients, mechanisms, and targets of the constant therapy program, conceptualized within the rehabilitation treatment specification system (RTSS) framework.

Constant Therapy (CT) is an app-based, evidence-based digital therapeutic designed to improve multiple domains of language simultaneously using a self-managed approach (www.constanttherapy.com) (33). Figure 2 depicts the CT therapy program using a tripartite schema (i.e., therapy target(s), ingredients, and mechanisms of action) following the Rehabilitation Treatment Specification System (RTSS) (34). There are several unique ingredients of the program, including (1) task variety with 266 different task types spanning speech, language and cognitive domains and functional daily activities that encompass them (e.g., listening to a voicemail, reading a map); (2) personalized goal setting enabling patients and their clinicians to identify high-priority, functionally relevant therapy goals across multiple domains; (3) adaptive difficulty that enables self-paced progression from easier to harder tasks within each targeted domain using an algorithm based on performance accuracy and consistency, allowing for therapy scaffolding in a way that mirrors in-person therapy techniques employed by skilled clinicians; (4) consistent feedback that is provided to the patient after every item, therapy goal and session; (5) ease of access that allows patients to log in and practice therapy at their convenience and progress at their own pace; and (6) the recommended therapy regimen that can be self-managed, reducing the need for regular face-to-face interaction with a clinician. Preliminary studies of CT have indicated that it is effective in inducing improvements in language outcomes in chronic post-stroke aphasia (22, 35, 36).

For this study, we aggregated data across 13 different skill domains: (1) analytical, (2) arithmetic, (3) attention, (4) auditory comprehension, (5) auditory memory, (6) naming, (7) phonological processing, (8) production, (9) quantitative, (10) reading, (11) visual memory, (12) visuospatial skills and (13) writing. When using the Constant Therapy program, users select skill domains they wish to improve and are assigned tasks based on that selection by the algorithm. Task difficulty is adjusted per individual user using an adaptive algorithm, with more difficult tasks assigned once patients have demonstrated mastery of prior tasks assigned with a high accuracy. The order in which more difficult tasks are assigned is according to a universal task progression order per domain. The progression order is thus a serial ranking of tasks from least to most difficult. Determination of each domain's progression order was based on research evidence in consultation with speech-language pathologists (37). Patient progress is subdomain specific, so improvement in one domain does not affect the progression order of other domains the patient is practicing simultaneously. In this way, during a session, patients practice tasks in order of subsequent increasing progression orders. Additionally, if a patient fails to improve at one progression order, a lower-level task will be assigned to the patient in addition to the original task. The Constant Therapy app records all data for each session for this study including accuracy per trial, latency per trial, the progression order, timestamp, total exercises, and session duration.

Because users practice different task types at different levels of difficulty, it is not enough to evaluate the performance outcome using an accuracy metric alone. Instead, we derived a summative metric of performance accuracy that allows for comparison across different skill domains and task difficulty levels, called domain score. In a specific session, the highest progression order of the task passed or worked on and the lowest progression order of the task failed are recorded. Here passing a task indicates accuracy of the task is equal to or greater than 90%, working on means more than 40% and less than 90%, while failure means accuracy is lower than 40%. The domain score of the session is calculated by averaging the two progression numbers, which is an estimate of the session's difficulty level. After that, the domain score is normalized by dividing it by the total number of progression orders in the specific domain. Normalization is required because the numbers of progression orders vary from domain to domain, and the original number alone cannot be used to compare directly across different domains. More details of domain score and its calculation have been previously described (23). By averaging the domain score across sessions in a week (only if there are multiple sessions in a single week), it is possible to evaluate the improvement or deterioration of patients' performance over time in a single domain.

### 2.3. Determination of the different dosage groups

Prior to discussing the data analyses, it is important to describe the determination of the different dosage groups. For a specific patient, the term exercise week indicates a week in which the patient has exercise records; unless explicitly noted, *week* is defined as exercise week in this study. In an *n*-week time period, the average dosage amount is calculated by summing up the dosage amount in the *n* exercise weeks and dividing it by the total number of calendar weeks the patient spent to complete *n* weeks of practice, which may include some additional weeks that do not have exercise records. Patients were then binned into the following three groups based on their average dosage amount over a period spanning 10 exercise weeks: 0–15 min per week (low dosage group), 15–40 min per week (medium dosage group), and more than 40 min per week (moderate dosage groups). It should be noted here that users practicing greater than 40 min per week on average demonstrated a large dosage range (up to 1,736 min per week in a 10-week period).

We considered 30 (exercise) weeks of time in total to evaluate the relationship between dosage amount and performance outcome. The 30-week period was split into three 10-week periods, and dosage amounts were averaged separately in the three periods. Patients were considered consistent (Analysis 1) only if (1) they had at least 30 exercise weeks on record and (2) for each of the 10-week time periods, they stayed within the same dosage group. Since this dataset is relatively small and not reflective of the more variable practice patterns that characterize the majority of app users, we also wanted to include an analysis of patients with more variable usage habits (Analysis 2). In the three 10-week periods, patients were included if they had practice records in each of the 10 weeks. Crucially for this analysis, a specific patient could appear in different groups in different time periods (e.g., 0–15 min/week group in the first 10 weeks vs. 15–40 min/week group in the second 10 weeks), so it is not possible to compare the same dosage amount group across multiple 10-week periods, hence data in the three time periods were analyzed separately.

### 2.4. Statistical analyses

For all statistical analyses, the first week of the therapy within a 10-week period of exercises was indicated as the baseline week, and a comparison of domain scores between later weeks and the baseline week was made to address the performance outcome over this 10-week period. Because we were primarily interested in the effect of dosage amount on performance outcome, we began by grouping patients according to their average weekly dosage amount, measured by calculating the mean minutes per week of therapy. Patients were then binned into one of the three groups introduced above: 0–15 min per week, 15–40 min per week, and more than 40 min per week.

Linear mixed-effect models (LMMs) were run in order to examine domain score changes over time as a function of dosage amount group. The weekly domain score served as the dependent variable in the model, with fixed effects of time (week number), dosage amount group, cumulative practice amount (i.e., total hours spent completing therapy tasks), time × dosage amount group, and time × cumulative practice amount. Covariates of age, time since stroke (≤6 and >6 months), sex, and baseline domain scores were also included as fixed effects in the model. The model included random effects of patients and domains.

All statistical analyses were conducted in R (version 4.1.2; R Foundation for Statistical Computing) using *lme4*, *lmerTest*, *emmeans*, and *sjPlot* packages.

### 2.5. Ethics approval

This project was considered an institutional review board–exempt retrospective analysis by Pearl Institutional Review Board (#17-LNCO-101) under 45 Code of Federal Regulations 46.101(b) category 2.

## 3. Results

### 3.1. Analysis 1: consistent users

A total of 537 patients and 1,448 records in different domains were selected as consistent practice patients by the criteria mentioned previously. As we are considering records of different domains from one specific patient separately, this can yield multiple records per patient. The statistical analysis is based on the total number of 1,448 records. Among these records 820 are from male patients while 628 records are from female patients. The average age of patients is 63.13 (SD, 13.68) years old with 48.7% (705) in the acute recovery stage (less than 6 months prior to therapy initiation). The summary statistics for the entire cohort and for each dosage amount group are presented in Table 1. In general, age, sex, and chronicity did not differ among dosage groups (*P* > .05 in all comparisons).

**Figure 3:**
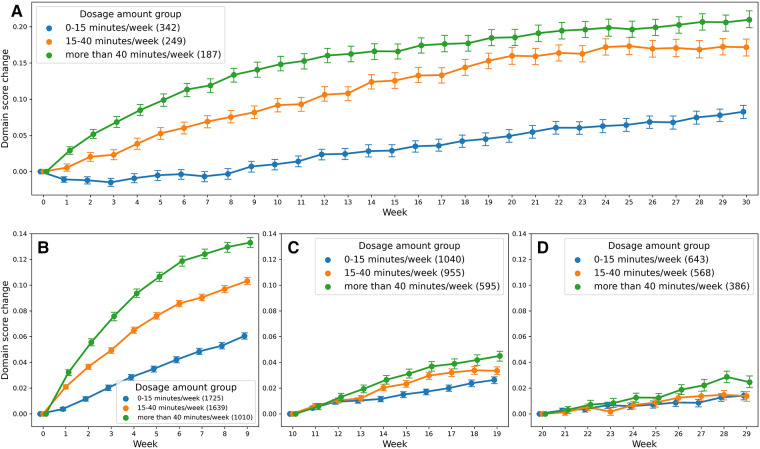
Change in domain score as a function of dosage amount group: (**A**) shows the score change for the consistent cohort, and (**B–D**) for the variable cohort.

Analysis 1 asked the question of whether greater average weekly dosage—defined as number of minutes per week—leads to greater performance gains over a 30-week period in a cohort of consistent users who practice approximately the same average amount week-to-week. The overall change in domain score (collapsed across domains) for the consistent group over 30 weeks is plotted in Figure 3A. The plot shows that, while all patients show improvements in the overall domain score, the 40+ min/week group shows greater changes in the domain score than the 0–15 min/week and 15–40 min/week groups over the 30-week time period. The statistical results for the consistent cohort are shown in Tables 2, 3. Specifically, a higher weekly domain score was associated with an increase in the number of weeks of therapy (*β *=* *.004; *t* = 6.09; *P* < .001), higher baseline domain score (*β *=* *.378; *t* = 67.74; *P* < .001), and greater practice amount (15–40 min/week: *β *=* *.034, *t* = 7.49, *P* < .001; 40+ min/week: *β *=* *.091, *t* = 14.70, *P* < .001). In addition, age (*β *=* *−.001; *t* = −2.80; *P* = .005) and time since stroke (*β *=* *.019; *t* = 2.08; *P* = .038) were also significant predictors of domain score, with younger age and acute chronicity associated with a higher weekly domain score. Sex was not a significant predictor of domain score.

**Table 2:** Final linear mixed-effects model results summary of consistent cohort (fixed effects).

Predictors	Estimates (SE)	*t* test (df)	*P* value
**Fixed effects**			
Intercept	2.51 × 10^−1^ (2.94 × 10^−2^)	8.56 (7.60 × 10^1^)	***
Week	3.88 × 10^−3^ (6.37 × 10^−4^)	6.09 (2.15 × 10^1^)	***
Dosage group (15–40 min/week)	3.43 × 10^−2^ (4.58 × 10^−3^)	7.49 (1.76 × 10^4^)	***
Dosage group (>40 min/week)	9.06 × 10^−2^ (6.17 × 10^−3^)	14.70 (9.37 × 10^3^)	***
Domain score baseline	3.78 × 10^−1^ (5.58 × 10^−3^)	67.74 (4.26 × 10^4^)	***
Age	−9.42 × 10^−4^ (3.37 × 10^−4^)	−2.80 (5.03 × 10^2^)	**
Sex (male)	3.94 × 10^−4^ (9.04 × 10^−3^)	0.04 (5.04 × 10^2^)	
Chronicity (acute)	1.86 × 10^−2^ (8.94 × 10^−3^)	2.08 (5.04 × 10^2^)	*
Week: Dosage group (15–40 min/week)	2.01 × 10^−3^ (2.63 × 10^−4^)	7.64 (1.77 × 10^4^)	***
Week: Dosage group (>40 min/week)	2.81 × 10^−3^ (3.54 × 10^−4^)	7.94 (9.30 × 10^3^)	***

Signif. codes: 0 “***” 0.001 “**” 0.01 “*” 0.05 “.” 0.1 “ ” 1.

Model equation: domain score (weekly average) ∼ 1 + week * dosage group + domain score baseline + age + sex + chronicity + (1 + week:patient) + (1 + week:domain).

**Table 3:** Final linear mixed-effects model results summary of consistent cohort (random effects).

Predictors	Variance (SD)	Correlation
**Random effects**		
Residual	1.65 × 10^−2^ (1.28 × 10^−1^)	N/A
Patient (intercept)	1.03 × 10^−2^ (1.02 × 10^−1^)	N/A
Domain (intercept)	4.46 × 10^−3^ (6.68 × 10^−2^)	N/A
Week:patient (slope)	3.78 × 10^−5^ (6.15 × 10^−3^)	−0.20
Week:domain (slope)	3.99 × 10^−6^ (2.00 × 10^−3^)	−0.34

Most importantly given our study objectives, the time × dosage amount group interaction was significant (*F* = 38.78, *P* < .001). From this result, we note that although all groups of consistent app users improved over the 30-week therapy period, the rate of improvement was driven by the weekly dosage amount. Compared to the group practicing 0–15 min per week, the 15–40 min per week group (*β *=* *.002, *t*_17,700 _= 7.64, *P* < .001) and the group practicing more than 40 min per week (*β *=* *.003, *t*_9,297 _= 7.94, *P* < .001) showed significantly higher weekly domain scores over time. A *post hoc* comparison of slopes across each of the three dosage groups revealed a significantly greater rate of improvement for the moderate dosage (40+ min/week) group compared to the medium dosage (15–40 min/week) group and the low dosage (0–15 min/week). This reinforces the notion that incremental increases in weekly therapy dosage (i.e., 0–15 vs. 15–40 vs. 40+ min/week) yield significantly greater gains in improvements over a 30-week period for this cohort of consistent app users (Table 4).

**Table 4:** Pairwise comparisons of slopes by dosage amount group (consistent cohort).

Contrast	Estimate (SE)	*t* test	*P* value
0–15 min/week vs. 15–40 min/week	−2.01 × 10^−3^ (2.63 × 10^−4^)	−7.64	***
0–15 min/week vs. >40 min/week	−2.81 × 10^−3^ (3.54 × 10^−4^)	−7.94	***
15–40 min/week vs. >40 min/week	−7.99 × 10^−4^ (2.94 × 10^−4^)	−2.69	*

Signif. codes: 0 “***” 0.001 “**” 0.01 “*” 0.05 “.” 0.1 “ ” 1.

Analysis 1 took a conservative approach to evaluate the effects of practicing long-term therapy, only users that consistently practiced for 30 weeks were included in the analyses. Consequently, the number of such users was relatively low, with only 537 individual users. A perusal of the database of users indicated that users were more likely to be variable in their practice patterns, sometimes practicing more often and sometimes practicing less often. To evaluate whether this variable practice pattern influenced the extent of domain score change, we conducted Analysis 2.

### 3.2. Analysis 2: variable users

In Analysis 2, in each 10-week period, the numbers of patients in this cohort are subject to change and vary from period to period. Demographic information about users in each of the three time periods is listed in Table 5. Age and sex are distributed evenly across the three time periods and the three groups in each time slot. However, the average baseline domain score increased over time (week 1–10: 0.33, week 11–20: 0.41, week 21–30: 0.43), which indicates that patients were improving as part of the continued therapy process. Another factor to note is that as time progressed, the portion of acute patients decreased (week 1–10: 57.0%, week 11–20: 53.1%, week 21–30: 48.3%).

**Table 5:** Summary statistics of the variable cohort.

Time period	Characteristics	Overall	0–15 min/week	15–40 min/week	>40 min/week
Week 1–10 (*N =* 12,112)	Age, mean (SD)	63.36 (13.51)	62.52 (13.77)	63.85 (13.71)	64.45 (12.35)
	Baseline domain score, mean (SD)	0.33 (0.20)	0.32 (0.21)	0.33 (0.19)	0.36 (0.18)
	**Sex, *n* (%)**				
	Male	6,902 (57.0)	3,061 (55.5)	2,488 (58.3)	1,353 (58.0)
	Female	5,151 (42.5)	2,423 (43.9)	1,764 (41.3)	964 (41.3)
	Other	59 (0.5)	30 (0.6)	12 (0.4)	17 (0.7)
	**Chronicity, *n* (%)**				
	Acute (<6 months)	6,899 (57.0)	3,080 (55.9)	2,468 (57.9)	1,351 (57.9)
	Chronic (>6 months)	5,213 (43.0)	2,434 (44.1)	1,796 (42.1)	983 (42.1)
Week 11–20 (*N =* 6,888)	Age, mean (SD)	63.39 (13.53)	62.59 (14.18)	63.99 (13.05)	64.45 (12.46)
	Baseline domain score, mean (SD)	0.41 (0.22)	0.37 (0.22)	0.43 (0.22)	0.47 (0.20)
	**Sex, *n* (%)**				
	Male	3,906 (56.7)	1,931 (57.4)	1,237 (55.4)	738 (57.3)
	Female	2,975 (43.2)	1,435 (42.6)	993 (44.5)	547 (42.5)
	Other	7 (0.1)	1 (0.0)	3 (0.1)	3 (0.2)
	**Chronicity, *n* (%)**				
	Acute (<6 months)	3,656 (53.1)	1,781 (52.9)	1,205 (54.0)	670 (52.0)
	Chronic (>6 months)	3,232 (46.9)	1,586 (47.1)	1,028 (46.0)	618 (48.0)
Week 21–30 (*N =* 4,162)	Age, mean (SD)	63.66 (13.06)	63.00 (13.31)	64.07 (13.05)	64.61 (12.38)
	Baseline domain score, mean (SD)	0.43 (0.23)	0.39 (0.23)	0.45 (0.23)	0.50 (0.21)
	**Sex, *n* (%)**				
	Male	2,398 (57.6)	1,151 (57.0)	746 (56.8)	501 (60.4)
	Female	1,762 (42.3)	867 (42.9)	567 (43.1)	328 (39.6)
	Other	2 (0.1)	1 (0.1)	1 (0.1)	0 (0)
	**Chronicity, *n* (%)**				
	Acute (<6 months)	2,011 (48.3)	989 (49.0)	622 (47.3)	400 (48.3)
	Chronic (>6 months)	2,151 (51.7)	1,030 (51.0)	692 (52.7)	429 (51.7)

*N* (patient 0–15 min/week) = 2,159.

*N* (patient 15–40 min/week) = 1,314.

*N* (patient >40 min/week) = 812.

Analysis 2 asked the question of whether greater amounts of weekly therapy led to greater performance gains across three consecutive 10-week intervals (for a total of 30 weeks), in a larger cohort of more variable users. As shown in Tables 6, 7, similar to the consistent cohort, time (week 1–10: *β *=* *.009, *t* = 9.04, *P* < .001, week 11–20: *β *=* *.003, *t* = 5.56, *P* < .001, week 21–30: *β *=* *.002, *t* = 3.60, *P* < .001), acute condition (week 1–10: *β *=* *.009, *t* = 3.52, *P* < .001, week 11–20: *β *=* *.015, *t* = 6.10, *P* < .001, week 21–30: *β *=* *.010, *t* = 3.73, *P* < .001) and greater baseline domain score (week 1–10: *β *=* *.604, *t* = 234.45, *P* < .001, week 11–20: *β *=* *.779, *t* = 301.62, *P* < .001, week 21–30: *β *=* *.813, *t* = 270.21, *P* < .001) were also associated with greater weekly domain score within each of the 10 week analysis periods. Figures 3B–D shows the change in domain score of this cohort in three different time periods.

**Table 6:** Final linear mixed-effects model results summary of the variable cohort (fixed effects).

Time period	Predictors	Estimates (SE)	*t* test (*df*)	*P* value
**Fixed effects**				
Week 1–10	Intercept	1.51 × 10^−1^ (1.31 × 10^−2^)	11.49 (2.27 × 10^1^)	***
	Week	9.00 × 10^−3^ (9.96 × 10^−4^)	9.04 (1.57 × 10^1^)	***
	Dosage group (15–40 min/week)	2.21 × 10^−2^ (1.69 × 10^−3^)	13.07 (3.93 × 10^4^)	***
	Dosage group (>40 min/week)	4.16 × 10^−2^ (2.27 × 10^−3^)	18.31 (1.90 × 10^4^)	***
	Domain score baseline	6.05 × 10^−1^ (2.58 × 10^−3^)	234.45 (1.14 × 10^5^)	***
	Age	−2.70 × 10^−4^ (9.62 × 10^−5^)	−2.81 (1.72 × 10^3^)	**
	Sex (male)	4.49 × 10^−4^ (2.63 × 10^−3^)	0.17 (1.71 × 10^3^)	
	Sex (not specified)	2.12 × 10^−2^ (1.77 × 10^−2^)	1.20 (1.84 × 10^3^)	
	Chronicity (acute)	9.29 × 10^−3^ (2.64 × 10^−3^)	3.52 (1.70 × 10^3^)	***
	Week: Dosage group (15–40 min/week)	2.74 × 10^−3^ (3.18 × 10^−4^)	8.60 (4.18 × 10^4^)	***
	Week: Dosage group (>40 min/week)	5.58 × 10^−3^ (4.28 × 10^−4^)	13.02 (2.07 × 10^4^)	***
Week 11–20	Intercept	7.14 × 10^−2^ (1.01 × 10^−2^)	7.05 (4.45 × 10^1^)	***
	Week	2.94 × 10^−3^ (5.29 × 10^−4^)	5.56 (2.56 × 10^1^)	***
	Dosage group (15–40 min/week)	1.59 × 10^−3^ (5.12 × 10^−3^)	0.31 (1.26 × 10^4^)	
	Dosage group (>40 min/week)	−5.01 × 10^−3^ (6.63 × 10^−3^)	−0.76 (6.24 × 10^3^)	
	Domain score baseline	7.79 × 10^−1^ (2.58 × 10^−3^)	301.62 (4.94 × 10^4^)	***
	Age	−2.80 × 10^−4^ (8.98 × 10^−5^)	−3.11 (9.26 × 10^2^)	**
	Sex (male)	−1.03 × 10^−4^ (2.44 × 10^−3^)	−0.42 (9.28 × 10^2^)	
	Sex (not specified)	−1.90 × 10^−2^ (3.25 × 10^−2^)	−0.59 (1.23 × 10^3^)	
	Chronicity (acute)	1.48 × 10^−2^ (2.43 × 10^−3^)	6.10 (9.28 × 10^2^)	***
	Week: Dosage group (15–40 min/week)	1.57 × 10^−3^ (3.51 × 10^−4^)	4.48 (1.38 × 10^4^)	***
	Week: Dosage group (>40 min/week)	3.33 × 10^−3^ (4.58 × 10^−4^)	7.27 (7.14 × 10^3^)	***
Week 21–30	Intercept	6.78 × 10^−2^ (1.17 × 10^−2^)	5.82 (1.02 × 10^2^)	***
	Week	1.63 × 10^−3^ (4.53 × 10^−4^)	3.60 (4.09 × 10^1^)	***
	Dosage group (15–40 min/week)	−4.00 × 10^−3^ (1.01 × 10^−2^)	−0.40 (3.02 × 10^3^)	
	Dosage group (>40 min/week)	−2.64 × 10^−2^ (1.25 × 10^−2^)	−2.11 (1.41 × 10^3^)	*
	Domain score baseline	8.13 × 10^−1^ (3.01 × 10^−3^)	270.21 (2.69 × 10^4^)	***
	Age	−3.99 × 10^−4^ (1.04 × 10^−4^)	−3.84 (5.94 × 10^2^)	***
	Sex (male)	−2.89 × 10^−4^ (2.77 × 10^−3^)	−0.11 (5.74 × 10^2^)	
	Sex (not specified)	−2.07 × 10^−2^ (4.64 × 10^−2^)	−0.45 (1.09 × 10^3^)	
	Chronicity (acute)	1.03 × 10^−2^ (2.75 × 10^−3^)	3.73 (5.73 × 10^2^)	***
	Week: Dosage group (15–40 min/week)	7.29 × 10^−4^ (4.14 × 10^−4^)	1.76 (3.11 × 10^3^)	.
	Week: Dosage group (>40 min/week)	2.49 × 10^−3^ (5.14 × 10^−4^)	4.85 (1.48 × 10^3^)	***

Signif. codes: 0 “***” 0.001 “**” 0.01 “*” 0.05 “.” 0.1 “ ” 1.

Model equation: domain score (weekly average) ∼ 1 + week * dosage group + domain score baseline + age + sex + chronicity + (1 + week:patient) + (1 + week:domain).

**Table 7:** Final linear mixed-effects model results summary of variable cohort (random effects).

Time period	Predictors	Variance (SD)	Correlation
**Random effects**			
Week 1–10	Residual	1.45 × 10^−2^ (1.21 × 10^−1^)	N/A
	Patient (intercept)	2.57 × 10^−3^ (5.07 × 10^−2^)	N/A
	Domain (intercept)	1.70 × 10^−3^ (4.13 × 10^−2^)	N/A
	Week: patient (slope)	1.36 × 10^−4^ (1.16 × 10^−2^)	0.43
	Week: domain (slope)	1.14 × 10^−5^ (3.38 × 10^−3^)	−0.15
Week 11–20	Residual	1.02 × 10^−2^ (1.01 × 10^−1^)	N/A
	Patient (intercept)	5.58 × 10^−3^ (7.47 × 10^−2^)	N/A
	Domain (intercept)	6.69 × 10^−4^ (2.59 × 10^−2^)	N/A
	Week: patient (slope)	6.42 × 10^−5^ (8.01 × 10^−3^)	−0.91
	Week: domain (slope)	2.29 × 10^−6^ (1.51 × 10^−3^)	−0.50
Week 21–30	Residual	9.08 × 10^−3^ (9.53 × 10^−2^)	N/A
	Patient (intercept)	1.62 × 10^−2^ (1.27 × 10^−1^)	N/A
	Domain (intercept)	3.29 × 10^−4^ (1.81 × 10^−2^)	N/A
	Week: patient (slope)	4.72 × 10^−5^ (6.87 × 10^−3^)	−0.98
	Week: domain (slope)	9.73 × 10^−7^ (9.87 × 10^−4^)	−0.56

Model equation: domain score (weekly average) ∼ 1 + week * dosage group + domain score baseline + age + sex + chronicity + (1 + week:patient) + (1 + week:domain).

Crucial to our question of interest, the interaction of time × dosage amount group was significant across each of the three 10 week analysis periods. Compared to the 0–15 min/week group, the 15–40 min/week (week 1–10: *β *=* *.003, *t* = 8.60, *P* < .001, week 11–20: *β *=* *.002, *t* = 4.48, *P* < .001) and 40 + min/week groups (week 1–10: *β *=* *.006, *t* = 13.02, *P* < .001, week 11–20: *β *=* *.3, *t* = 7.27, *P* < .001) showed greater rates of performance improvement in the first and second 10-week analysis intervals. Post hoc comparisons of slopes (Table 8) demonstrated a significantly greater rate of improvement also for the 40 + min/week compared to the 15–40 min/week in both the first and second 10-week intervals. For the final 10-week analysis interval (i.e., weeks 20–29 of therapy), a similar pattern of significance emerged, with the rate of improvement being significantly greater for 40+ min/week vs. 0–15 min/week group (*β *=* *.002, *t* = 4.85, *P* < .001), but with no significant difference in rates of improvement for the 15–40 and 0–15 min/week (*β *=* *.001, *t* = 1.76, *P* = .078). Post hoc tests revealed there was also a significantly greater rate of improvement for the moderate vs. medium dosage group.

**Table 8:** Pairwise comparisons of slopes by dosage amount group (variable cohort).

Time period	Contrast	Estimate (SE)	*t* test	*P* value
Week 1–10	0–15 min/week vs. 15–40 min/week	−2.74 × 10^−3^ (3.18 × 10^−4^)	−8.60	***
	0–15 min/week vs. >40 min/week	−5.58 × 10^−3^ (4.28 × 10^−4^)	−13.02	***
	15–40 min/week vs. >40 min/week	−2.84 × 10^−3^ (3.89 × 10^−4^)	−7.30	***
Week 11–20	0–15 min/week vs. 15–40 min/week	−1.57 × 10^−3^ (3.51 × 10^−4^)	−4.48	***
	0–15 min/week vs. >40 min/week	−3.33 × 10^−3^ (4.58 × 10^−4^)	−7.27	***
	15–40 min/week vs. >40 min/week	−1.75 × 10^−3^ (4.32 × 10^−4^)	−4.06	***
Week 21–30	0–15 min/week vs. 15–40 min/week	−7.29 × 10^−4^ (4.14 × 10^−4^)	−1.76	
	0–15 min/week vs. >40 min/week	−2.49 × 10^−3^ (5.14 × 10^−4^)	−4.85	***
	15–40 min/week vs. >40 min/week	−1.76 × 10^−3^ (5.07 × 10^−4^)	−3.48	**

Signif. codes: 0 “***” 0.001 “**” 0.01 “*” 0.05 “.” 0.1 “ ” 1.

## 4. Discussion

This study aimed to examine if self-managed therapy could be sustained over a long period of time, and if greater average amounts of therapy were associated with greater therapy outcomes. To address these broad questions, therapy practice over a course of a 30-week treatment period (i.e., 6 months) was evaluated for different dosage amounts. Specifically, we evaluated whether greater average weekly dosage—defined as number of minutes per week—led to greater performance gains over a 30-week period in a cohort of consistent users who practice approximately the same average amount week to week. A second analysis examined a larger cohort of variable users, also over a course of 30-week period, to see if performance outcomes at each 10-week period showed relative greater gains for high practice frequency than lower practice frequencies.

**Figure 4:**
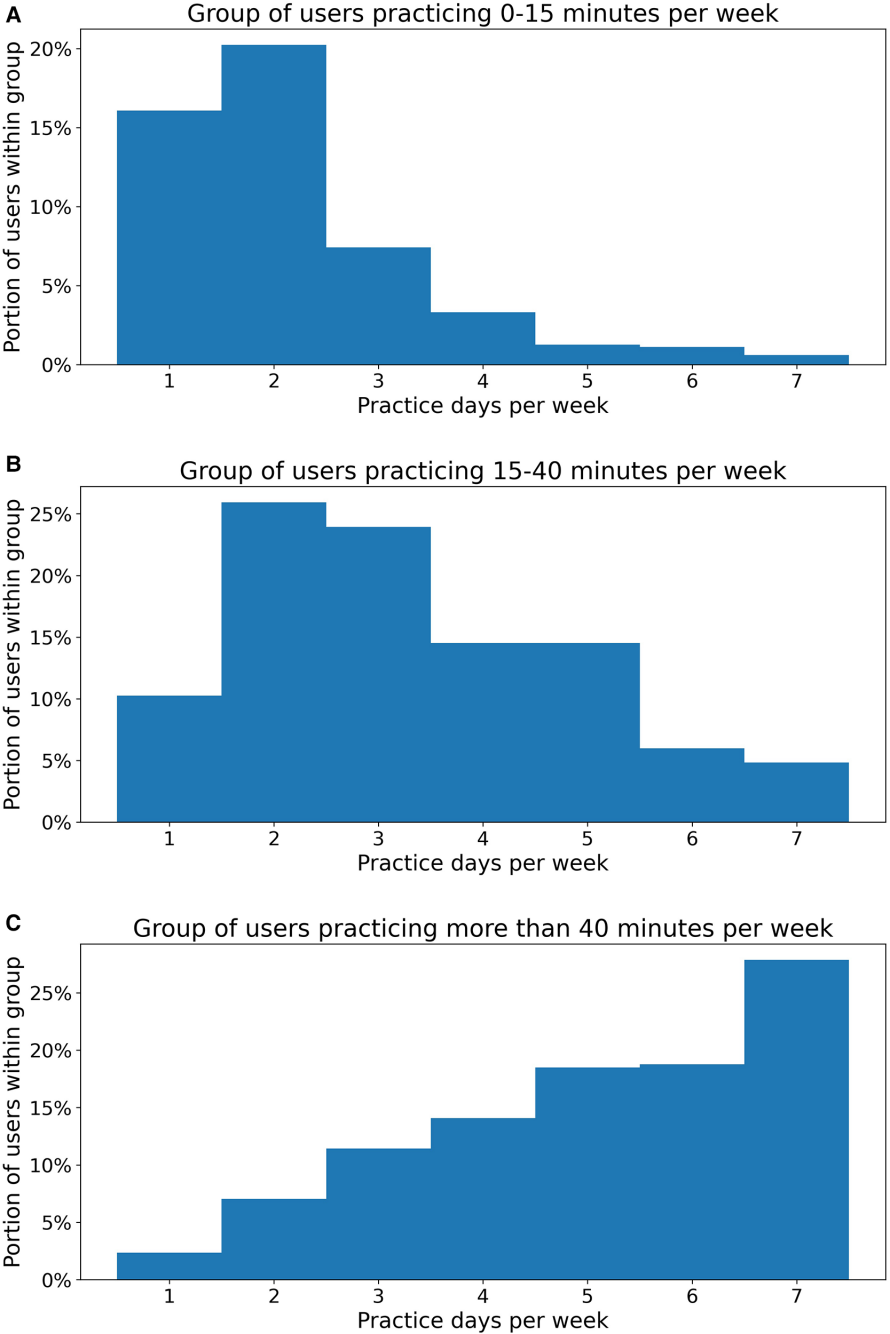
Practice frequency distribution of users in different dosage amount groups.

There were several main conclusions to be drawn from our study results. Firstly, patients were able to practice consistently for 30 weeks of self-managed therapy and this practice was associated with concurrent improvements in domain scores. Not surprisingly, in this context, users who practiced more than 40 min per week showed greater improvements in the average domain score than users who practiced less than 15 min per week. These results suggest that consistent and sustained practice can result in therapy improvements and that these gains are maintained 20–30 weeks from the therapy onset time. Notably, patients who practiced more variably over a 30-week treatment period likewise demonstrated that greater weekly average dosage amounts were associated with greater improvements in overall domain score. In particular, users who practiced more than 40 min per week showed significantly greater performance gains than users who followed a medium (15–40 min) or low (0–15 min) dosage practice regimen. This was the case in each of the three 10-week intervals of interest, demonstrating that dosage amount matters for therapy outcomes not just in the beginning but also throughout the course of treatment. It should also be noted, as Figure 4 shows, users who practiced more than 40 min per week (Figure 4C) also tended to practice more frequently, with a portion of 65.1% practicing more than 5 days per week and 27.8% practiced every day, compared to less frequent, more massed practice patterns in the medium (15–40 min) (Figure 4B) and low (0–15 min) (Figure 4A) dosage groups.

One notable observation is that by the 21–30 week period, the proportion of chronic patients (greater than 6 months post injury) was higher than in the first 1–10 week period, where they were more acute patients (less than 6 months post injury). This observation was true for both the consistent and variable group analyses. These results suggest that chronic survivors are able to sustain practice over long periods of time (>20 weeks) and demonstrate noticeable improvements on the domain score within the Constant Therapy program.

Results from both consistent and variable user cohorts demonstrated significant gains in domain score across the entire 30-week period of interest in our analyses. In both cohorts, the greatest rates of improvement occurred in the early weeks of therapy but crucially, all users were able to maintain performance gains during later weeks of therapy (e.g., weeks 10–20; 20–30). Moreover, for users following a relatively higher dosage practice regimen, these additional weeks of therapy resulted not only in maintenance of initial gains but in significant additional gains. This result underscores the promise of higher dose therapy to induce gains over a much longer time period than has previously been shown.

In line with prior research, our results show that relatively higher dosage therapy regimens are associated with greater gains in performance as compared to medium or low dosage regimens (6, 38). This study is among a relatively small number of studies to directly compare the effects of varied dose of the same behavioral intervention. The small number of these dose articulation studies has been identified as a major barrier to the development of optimal dosage guidelines for speech-language pathologists. A recent systematic review found just six studies that reported direct dosage comparisons, and all of these focused on traditional clinician-mediated interventions (39). To our knowledge, only one prior study has investigated the effect of varied dosage on treatment outcomes for a self-managed digital therapy (23). The current study extends on this finding in critical ways by demonstrating that (1) high-intensity, self-managed SLT leads to significant performance gains over a much more extended therapy time than previously shown (30 vs. 10 weeks) and (2) performance gains are greater for users who practice a greater average amount, regardless of whether they are consistent or more variable in their usage pattern.

The current study also contributes to existing literature through its use of a real-world, ecologically valid dataset. Although the efficacy of high dose speech-language therapy has been established in the literature, there is a gap in translating these research findings to clinical practice. Translation of research findings is complicated by several barriers that include, among others, a large discrepancy in the amount of therapy recommended in research compared to the amount of therapy that is realistically attainable in the clinical setting (15, 18). By analyzing data from two cohorts of patient users who showed natural divergence in the pattern and amount of app-based practice logged over the 30-week time period, we were able to investigate effects of different dosage amounts taking into account the actual amount and types of practice of a large number of real-world users. This ensures greater generalizability of results to the clinical and real-world settings. Our results are encouraging because they not only show that higher intensity (40 min or more per week in our study) is feasible for a sizable group of real-world users but they also show that regardless of your exact pattern of practice (consistent vs. variable; moderate vs. medium vs. low), self-managed SLT leads to significant and sustained performance gains. Our results also demonstrate that higher-intensity therapy may look different in self-managed settings compared to highly controlled laboratory or clinical trial settings. In the latter, weekly dosage prescriptions are very high but total intervention duration is relatively short, whereas in our data, weekly dosage amounts are comparatively more modest but users instead practice for many more weeks (30+ weeks), resulting in cumulative dosage amounts that are comparable to high-intensity regimens as reported in the literature (38). Also important to consider is that CT or other app-based, at-home therapy can be used as an adjuvant to other SLT within the context of patients' longer-term trajectory of recovery; patients may for instance receive direct SLT in early post-acute recovery stages but turn to use of at-home, self-managed therapy after exhausting options for insurance-covered direct SLT. Finally, we note that the data analyzed in this study is the result of entirely self-managed practice, meaning that users were not given explicit instructions on the amount or frequency with which to practice. It is likely that dosage amounts—and possibly also the resultant therapy gains—could be augmented if users were advised on a specific practice regimen.

Importantly, the current study focused on measuring improvement *via* an in-app task improvement measure (i.e., domain score). Though outside the scope of the current study, it will be essential in future work to evaluate the generalizability of in-app domain score improvements to standardized measures of global language severity (e.g., WAB-R Aphasia Quotient), to real-world communication settings and conducted with large numbers of users. Prior clinical studies of the Constant Therapy app have reported clinically significant gains in both global language severity measures and quality of life scores following in-app practice (35, 36). Des Roches et al. found significant pre-post improvements on the WAB-R Aphasia Quotient and composite severity score on the Cognitive Linguistic Quick Test among an experimental group of patients using the CT program as an adjuvant to traditional SLT; no such changes were seen among control participants receiving only traditional SLT (36). Most recently, Braley and colleagues conducted a randomized clinical trial comparing language-based outcomes following digital-only CT therapy compared to traditional SLT. Participants receiving digital-only CT therapy improved 6.75 points on the WAB-R AQ and also demonstrated significant improvement in overall quality of life, as measured by the Stroke and Aphasia Quality of Life Scale 39 (SAQOL-39) (35). Taken together, these findings lend encouraging evidence in support of treatment generalization for CT app users. It is also worthwhile to note that unlike rote paper-and-pencil therapy exercises, CT tasks are functional in nature (e.g., reading a museum map to determine where a given exhibit is), which may make it more likely for in-app improvements to generalize to out-of-app settings.

### 4.1. Limitations

We note several limitations of the current study. First is the lack of standardized performance metrics to characterize baseline severity and relatedly, the reliance on patient self-report for reporting of demographic and etiological details. The Constant Therapy app makes it possible to collect a large amount of real-world data about users and their daily performance patterns but because it is entirely self-managed, our dataset did not include standardized assessment metrics that might typically be collected in a clinic setting (e.g., Western Aphasia Battery-Revised aphasia quotient). Likewise, we did not have access to detailed information about concurrent medical and cognitive comorbidities, motivation levels, or personality types, all of which have the potential to influence therapy outcomes. Our analysis models do take into account basic demographic information such as age, sex, and chronicity and we also include random effects of patients in all analysis models. For baseline severity, we use the baseline domain score as a proxy measure. Nonetheless, future models with more detailed patient factors would likely lead to more robust and generalizable results.

A second limitation of the current study relates to the way in which users were assigned to their respective dosage groups and the way in which we chose to bin these groups. Users were binned into one of the three dosage amount groups according to their usage patterns and not by random assignment, leading to the possibility for some degree of self-selection into these groups (e.g., more severe users practicing less). To account for this potential effect of severity on results, we included baseline domain score—our proxy for starting severity—as a covariate in all statistical models. We also acknowledge that the current study employs data-informed but clinically arbitrary cutoffs to determine grouping into low, medium and moderate dosage groups. We therefore are careful to interpret results as providing support for higher vs. lower dose therapy rather than for a specific therapy prescription in minutes (e.g., 40 or min/week).

A final limitation is that there is insufficient information available on whether users had access to other direct therapy services. It is possible that some users may have used the app-based regimen in combination with traditional, in-person SLT, while others may have solely relied on the app. Differences in the amount of outside (i.e., non-app-based) therapy received by users across the dosage groups could potentially affect the results. High dosage app users may also be receiving more outside therapy, making it difficult to attribute any improvement in performance solely to increased in-app practice.

## 5. Conclusion

This study explored the relationship between the weekly dosage amount that stroke patients practiced in an app-based, self-managed therapeutic program and their performance improvement over a 30-week period. The results showed that across all users, the moderate dosage group (more than 40 min per week) achieved greater performance gains compared to medium (15–40 min per week) and low (0–15 min per week) dosage groups. A similar trend was noted between the medium and low dosage groups. Thus, our results show that performance gains are greater for users who practice a greater average amount. One possible further research direction could be suggesting a new evaluation metric to link in-app performance gains with real-world improvement.

## Data Availability

The original contributions presented in the study are included in the article/Supplementary Materials, further inquiries can be directed to the corresponding author.
